# *Camellia sinensis* (L.) Kuntze Extract Attenuates Ovalbumin-Induced Allergic Asthma by Regulating Airway Inflammation and Mucus Hypersecretion

**DOI:** 10.3390/pharmaceutics15092355

**Published:** 2023-09-20

**Authors:** Sohi Kang, Hyun-Yong Kim, A Yeong Lee, Hyo Seon Kim, Jun Hong Park, Byeong Cheol Moon, Hyeon Hwa Nam, Sung-Wook Chae, Bokyung Jung, Changjong Moon, In Sik Shin, Joong Sun Kim, Yun-Soo Seo

**Affiliations:** 1 College of Veterinary Medicine and BK21 FOUR Program, Chonnam National University, Gwangju 61186, Republic of Korea; shrloveu@jnu.ac.kr (S.K.); bkjung@jnu.ac.kr (B.J.); moonc@jnu.ac.kr (C.M.); dvmmk79@gmail.com (I.S.S.); 2 Herbal Medicine Resources Research Center, Korea Institute of Oriental Medicine, 111, Geonjae-ro, Naju-si 58245, Jeollanam-do, Republic of Korea; khy9514@kiom.re.kr (H.-Y.K.); lay7709@kiom.re.kr (A.Y.L.); hs0320@kiom.re.kr (H.S.K.); jhpark@kiom.re.kr (J.H.P.); bcmoon@kiom.re.kr (B.C.M.); namhh@kiom.re.kr (H.H.N.); 3 Center for Companion Animal New Drug Development, Jeonbuk Branch, Korea Institute of Toxicology, Jeongeup 56212, Jeollabuk-do, Republic of Korea; kendall@kiom.re.kr; 4 KM Convergence Research Division, Korea Institute of Oriental Medicine, 111, Geonjae-ro, Naju-si 58245, Jeollanam-do, Republic of Korea

**Keywords:** *Camellia sinensis* (L.) Kuntze, eosinophilic inflammation, airway inflammation, asthma, mucus hypersecretion

## Abstract

Asthma is a pulmonary disease induced by the inhalation of aeroallergens and subsequent inappropriate immune responses. *Camellia sinensis* (L.) Kuntze has been evaluated as an effective antioxidant supplement produced from bioactive compounds, including flavonoids. In this study, we aimed to determine the effects of *Camellia sinensis* (L.) Kuntze extract (CE) on ovalbumin-induced allergic asthma. The components of CE were analyzed using high-performance liquid chromatography (HPLC) chromatogram patterns, and asthmatic animal models were induced via ovalbumin treatment. The antioxidant and anti-inflammatory effects of CE were evaluated using 2,2-diphenyl-1-picryl-hydrazyl-hydrate (DPPH), 2,2′-azino-bis-3-ethylbenzthiazoline-6-sulphonic acid (ABTS), and nitric oxide (NO) assays. Seven compounds were detected in the CE chromatogram. In the ovalbumin-induced mouse model, CE treatment significantly decreased the inflammation index in the lung tissue. CE also significantly decreased eosinophilia and the production of inflammatory cytokines and OVA-specific IgE in animals with asthma. Collectively, our results indicate that CE has anti-inflammatory and antioxidant activities, and that CE treatment suppresses asthmatic progression, including mucin accumulation, inflammation, and OVA-specific IgE production.

## 1. Introduction

Asthma is a type of major respiratory disease that impacts quality of life, with different disease patterns observed in children and adults relating to its point of incidence and prevalence [1]. Almost twenty-five thousand people still die because of asthma each year according to WHO research [2]. This disease occurs due to heterogeneous, complex gene interaction, and 330 million people suffered from decreased productivity with a heavy cost. The characterization of asthma was related to the sensitization of allergies in children and polysensitization in an adult group [3].

Immune system activation, which is regulated by genetic and environmental triggers, is the main orchestrator of asthma worldwide [4]. Asthma is a chronic inflammatory disease of the airways and is exacerbated by a viral infection. Indeed, COVID-19 infection was assumed to exacerbate the risk of asthmatic symptoms [5]. Previous research has shown that disease progression worsened in children with a comorbidity of COVID-19 and asthma, and luteolin, the drug considered a potential therapeutic against asthma, will be a critical candidate for COVID-19/asthma comorbidity [6]. Furthermore, there are many comorbidities associated with asthma, including rhinitis, sinusitis, and hormone disorder, and they share similar pathophysiological process with asthma [7].

Cytokines promote chronic airway inflammation by promoting intercellular interaction, and T-helper-2 cells and type 2 innate lymphoid cells are representative cells that produce interleukin (IL)-4, -5, and -13 [8]. Inflammatory cytokines such as interleukin (IL)-4, -5, and -13 initiate the immunopathological action of asthma, causing eosinophilic inflammation, the synthesis of immunoglobulin (Ig)E and chemokines, mucus production, and inflammatory cell infiltration [9,10]. For this reason, there have been trials of developed biological anti-interleukin therapy in asthma-targeted IL-4, -5, and -13, with cases of success [11]. Although inhaled corticosteroids and beta2 agonists are used to treat asthma, they can reduce airway inflammation and relax smooth muscle, but the condition worsens since its fundamental cause remains unchanged. Previous studies have used steroids that block immune responses to treat asthma and reduce asthma symptoms; however, these drugs have limited application owing to their side effects and weak efficacy [12]. Thus, there is increasing demand for the development of medicines that are highly effective and have minimal side effects to successfully manage asthma.

Parts of the *Camellia sinensis* plant (leave, bud, and stalk) have been used to prepare tea products, and their biologically active components have been studied through a lot of research [13]. Among them, the green tea plant (*Camellia sinensis* (L.) Kuntze) is a commercial plant that has perennial and monoculture features, while the *Camellia* genus is reported to be the largest genus in the Theaceae family [14]. *C. sinensis* (L.) Kuntze has been evaluated as an effective supplement to prevent obesity [15] and treat vascular disease [16], and as a component of cosmetics to reduce skin damage [17]. The antioxidant effects of *C. sinensis* (L.) Kuntze have been proven through various antioxidant in vitro assays [18], while it has also been shown to exhibit antidiabetic [19], hepatoprotective [20], and component-level antibacterial effects [21]. Flavan-3-ol components have been identified, including gallocatechin, catechin, and epicatechin, in *C. sinensis* (L.) Kuntze extract, and these components showed outstanding bioactivity in a previous study [22]. A fermentation analysis of tea leaf compounds revealed that flavanols and flavonols were the primary bioactive compounds, while *C. sinensis* (L.) Kuntze and its products contained theaflavins. In addition, these compounds have demonstrated anti-inflammatory, antioxidant, and anticancer properties [19].

In addition, the therapeutic benefits of green tea have been reported in several types of lung disease models, including chronic obstructive pulmonary disease [23], smoking [24,25], and lung cancer [26]. However, the effects of green tea on asthma remain controversial. In this study, we used an ovalbumin (OVA)-induced asthma model to investigate the effects of *C. sinensis* (L.) Kuntze extract (CE) on asthmatic disease and metabolism.

## 2. Materials and Methods

### 2.1. Plant Materials

The leaves of green tea, *C. sinensis* (L.) Kuntze, were grown at and purchased from a herbal medicine store in Boseong-gun, Jeonnam, Republic of Korea. Sungyu Yang and Goya Choi at the Korea Institute of Oriental Medicine (KIOM) carried out the morphological verification of herbs, and the voucher specimen was deposited in the Korean Herbarium of Standard Herbal Resources (No. 2-21-0064).

### 2.2. Extraction Procedure and Chemicals

A total of 1 kg of *C. sinensis* (CS) was mixed using a blender (HMF-3000S; Hanil electric, Seoul, Republic of Korea), before ultrasonicating twice in an ultrasonic bath (Branson 8800 model, Branson Ultrasonic Corporation, Danbury, CT, USA) containing 5 L of 70% ethanol for 1 h. Subsequently, the extract was filtered using chromatography paper (46 cm × 57 cm), and the solvent was removed using a rotary evaporator. The yield of CE was 211.9 g (21.19%. *w*/*w*), which was stored at −20 °C. (−)-Epigallocatechin (EGC), (−)-epigallocatechin gallate (EGCG), and caffeine were purchased from Wako (Fujifilm Wako Pure Chemicals Ltd., Osaka, Japan); (+)-catechin was purchased from Sigma-Aldrich (Merck KGaA, Darmstadt, Germany); and epicatechin (EC), epicatechin gallate (ECG), and (−)-gallocatechin (GC) were obtained from ChemFaces (Wuhan, Hubei, China). High-performance liquid chromatography (HPLC)-grade formic acid, methanol, and acetonitrile were acquired from J.T. Baker products (Avantor Performance Materials Korea Ltd., Suwon, Republic of Korea), and LC–MS-grade distilled water was purchased from Merck (Merck KGaA, Darmstadt, Germany).

### 2.3. High-Performance Liquid Chromatography (HPLC) Analysis

A total of 82.93 mg of CE was dissolved in 4 mL of 70% ethanol and filtered through a 0.2 μm syringe filter before high-performance liquid chromatography (HPLC) analysis. All standard components were dissolved in 70% ethanol to a concentration of 100 μg/mL. The HPLC system (Waters Corporation, Milford, MA, USA) consisted of an Acquity Quadrupole Daltone mass (QDa) detector, a separation module (Waters e2695), a 2998 PDA detector (Waters), and a micro-splitter (IDEX Health & Science LLC, Oak Harbor, WA, USA). Eight small molecules in CE were separated using Luna 3u Phenyl-Hexyl (150 mm × 3.0 mm, 3.0 μm; Phenomenex Distributor, SungMoon Systech Corp, Seoul, Republic of Korea). The column and auto-sampler temperatures were 35 °C and 10 °C, respectively. The injection volume and flow rate were 3 μL and 0.4 mL/min, respectively. The PDA detection wavelength was monitored from 200 to 420 nm, and the targeted peaks were detected at 270 nm. The mobile phase was 0.05% aqueous formic acid (A), methanol (B), and 0.05% aqueous acetonitrile (C), and the linear gradient program was as follows: 90% A to 85% A (4% B and 6% C to 6% B and 9% B) for 0–2 min, and 85% A to 65% A (6% B and 9% C to 15% B and 20% C) for 2–30 min. The QDa was established as follows: nitrogen carrier gas, positive and negative TIC mode, 30–800 Da mass range, ESI capillary at 0.80 kV, probe temperature at 600 °C, con voltage of 15 V, source temperature at 120 °C, and a 5:1 split. Data analysis was performed using the Empower ver. 3 program (Waters), and sample peaks were confirmed by comparing the retention time, λmax of each compound, and molecular weight of the standard components.

### 2.4. Determination of Nitric Oxide (NO) Generation in RAW 264.7 Cells

The media concentrations of nitric oxide (NO) were examined using the Griess reagent kit (Promega, Madison, WI, USA). RAW 264.7 cells (1.0 × 10^4^/well) were precultured in 96-well plates, before treating with lipopolysaccharide (LPS; 1 μg/mL) at various concentrations of CE (25, 50, 100, and 200 μg/mL) for 24 h. NO production was measured at 540 nm and quantified using a sodium nitrite standard curve.

### 2.5. Antioxidant Assay

#### 2.5.1. 2,2-Diphenyl-1-picryl-hydrazyl-hydrate (DPPH) Radical Scavenging Assay

For the radical scavenging assay, 180 μL of 2,2-diphenyl-1-picryl-hydrazyl-hydrate (DPPH) solution was added to MEOH and 20 μL of each sample, before mixing in a 96-well plate and placing in the dark to react for 30 min at room temperature. Following incubation, the absorbance was measured at 570 nm using a microplate reader (SpectraMaxi3X, SpectraMax, Sunnyvale, CA, USA). Gallic acid was used as the positive control.

#### 2.5.2. 2,2-Diphenyl-1-picryl-hydrazyl-hydrate (ABTS) Radical Scavenging Assay

A total of 0.2 mM 2,2′-azino-bis-3-ethylbenzthiazoline-6-sulphonic acid (ABTS) diammonium salt was prepared in 3.5 mM potassium persulfate aqueous solution and diluted 10-fold in distilled water. ABTS radicals (ABTS+) were produced by keeping them in the dark for 14 h. Next, 10 μL of each sample was mixed with 290 μL of ABTS+ solution in a 96-well plate and incubated for a further 10 min in the dark. The absorbance was measured at 750 nm. Gallic acid was used as the positive control.

#### 2.5.3. Dichlorodihydrofluorescein-diacetate (DCFDA) Assay of the ROS-Scavenging Activity of the Aqueous Larval Extract

Dichlorodihydrofluorescein-diacetate (DCFDA), a fluorophore sensitive to intracellular ROS, was used to detect intracellular ROS production. OP9 cells on 35 mm cell culture dishes were treated with H_2_O_2_ with or without CE pretreatment for 30 min and cultured at 37 °C for 2 h. DCFDA solution (final concentration of 5 μM) was added to the suspension, and the mixture was incubated in the dark for 30 min at 37 °C, during which non-fluorescent DCFDA was oxidized to the highly fluorescent DCF in the presence of ROS [27]. Fluorescence intensity shifts were measured utilizing a fluorescence plate reader (Paradigm; Beckman Coulter, San Jose, CA, USA) at excitation and emission wavelengths of 485 and 530 nm, respectively.

### 2.6. Animals

BALB/c mice (6 weeks old) weighing 20 g were obtained from Samtako (Gyeonggi-do, Republic of Korea) and maintained at 22 ± 2 °C (humidity 55% ± 15%) for 1 week, with a 12 h/12 h light/dark cycle and food and water supplied ad libitum. After acclimatization and quarantine, the mice were divided into the following five groups: normal control (CON), ovalbumin-induced asthma group (OVA), ovalbumin-treated group using 5 mg/kg dexamethasone (DEX), ovalbumin-treated group with 100 mg/kg sample (CE100), and 200 mg/kg-treated group (CE200). All experimental procedures and the treatment of the animals were performed with the approval of the Animal Care Committee of Chonnam National University (CNU IACUC-YB-2022-33).

### 2.7. Experimental Procedures

The OVA-induced asthma model was generated by injecting 2 mg aluminum hydroxide and 20 μg OVA in phosphate-buffered saline (PBS) into BALB/c mice every 2 weeks. After the first injection (i.p.) of ovalbumin (OVA) solution, 1% OVA was administered through inhalation using an ultrasonic nebulizer (NE-U12, Omron Corp., Tokyo, Japan) for 30 min per day from days 21 to 23. Samples were orally administrated for 6 days (18–23 days) after the first OVA treatment at doses of 100 and 200 mg/kg. DEX (5 mg/kg) was administered as the control using the same method. Forty-eight hours following the final challenge, the animals were sacrificed via intraperitoneal injection of alfaxalone (85 mg/kg; Jurox Pty Ltd., Rutherford, NSW, Australia) and 10 mg/kg xylazine (Rompun^®^; Bayer Korea, Seoul, Republic of Korea), before performing tracheostomy. To conduct histological tests, the tracheas were cannulated and the left bronchi were tied. Bronchoalveolar lavage fluid (BALF) was collected after injecting 0.5 mL of ice-cold PBS into the lungs three times (total volume: 1.5 mL). The BALF was centrifuged (200× *g*, 10 min, 4 °C); the supernatant was kept at −20 °C for cytokine analysis; and the cell pellet was used to calculate the number of inflammatory cells.

### 2.8. Measurement of Inflammatory Cells in BALF

Alfaxalone (80 mg/kg, Jurox Pty Ltd., Rutherford, NSW, Australia) was used to anesthetize the mice, and tracheostomy was performed to collect BALF. Briefly, cold PBS (0.7 mL) was delivered into the lungs via injection into the trachea. The cells were transferred onto a slide using Cytospin (1500 rpm, 10 min; Hanil, Republic of Korea) following extraction of BALF from the animals. Each glass slide was stained with Diff-Quik solution (Sysmex, Horgen, Switzerland) to count the inflammatory cells.

### 2.9. Measurement of IL-4 and IgE Levels in BALF

BALF and plasma were collected and stored at −70 °C after centrifugation (300× *g*, 10 min). The levels of IL-4 and OVA-specific IgE were determined via an enzyme-linked immunosorbent assay (ELISA) (Mouse IL-4 DuoSet ELISA, DY404-05, R&D Systems, Minneapolis, MN, USA; Mouse IgE ELISA Kit, ab157718, Abcam, Cambridge, UK; LEGEND MAX™ Mouse OVA-Specific IgE ELISA Kit, BioLegend, San Diego, CA, USA). The IL-4 and OVA-specific IgE ELISA was performed according to the manufacturer’s protocol. Duplicate samples were diluted 1:100 in plasma. OVA-specific IgE levels in each sample were measured using optical density readings at 450 nm, and specific concentrations were calculated from a standard curve generated using recombinant IgE (5–2000 ng/mL).

### 2.10. Histology

After BALF isolation, the right lung tissues were removed and fixed in formalin at 4% (*v*/*v*); then they were dehydrated, embedded in Paraplast wax, and cut into 4 μm sections that were deparaffinized with xylene. Subsequently, the lung tissue sections were stained with hematoxylin and eosin (H&E) and periodic acid-Schiff (PAS; IMEB, San Marcos, CA, USA) to measure inflammation and mucus secretion, respectively. The stained sections were analyzed using a Motic Easyscan Digital Slide Scanner (Motic, Hong Kong, China). Quantification was conducted using a previously described image analysis approach [9].

### 2.11. Statistical Analysis

The results are expressed as the mean ± standard error of the mean (SEM). All statistical analyses were performed using GraphPad Prism 8.0 (GraphPad, San Diego, CA, USA). One-way ANOVA followed by Dunnett’s multiple comparison test was used to prove statistically significant differences between groups. Statistical significance was defined as *p* < 0.05.

## 3. Results

### 3.1. HPLC Analysis

**Figure 1:**
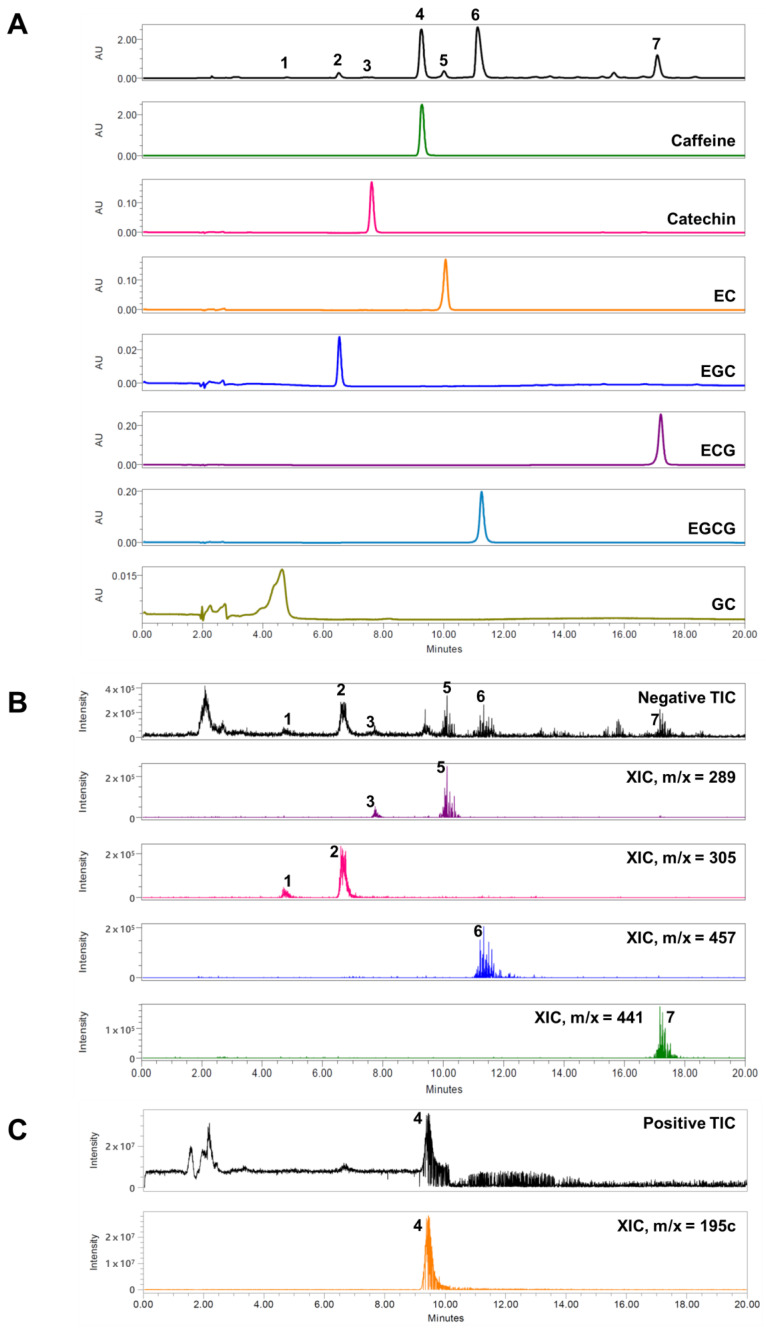
Representative HPLC chromatogram of CE at 270 nm (**A**), negative ion chromatogram (**B**), and positive ion chromatogram (**C**). 1. gallocatechin (GC); 2. epigallocatechin (EGC); 3. catechin; 4. caffeine; 5. epicatechin (EC); 6. epigallocatechin gallate (EGCG); and 7. epicatechin gallate (ECG). HPLC: high-performance liquid chromatography, CE: *Camellia sinensis* (L.) Kuntze extract.

The components of CE were quantified using HPLC. The analysis at 270 nm identified seven components of CE (Figure 1A), and the chromatogram was displayed for up to 20 min. Gallocatechin (GC) (at 4.6), epigallocatechin (EGC) (at 6.5), catechin (at 7.6), caffeine (at 9.3), epicatechin (EC) (at 10.1), epigallocatechin gallate (EGCG) (at 11.3), and epicatechin gallate (ECG) (at 17.2 min) were detected with λmax values of 270.0, 278.3, 267.6, 278.3, 273.6, and 277.1 nm, respectively (Figure 1A). In the negative mass mode, GC, EGC, catechin, EC, EGCG, and ECG were measured, where GC and EGC were [M − H]^−^ = 305.18 and 304.94 *m*/*z*, respectively; catechin and EC were detected as [M − H]^−^ = 289.20 and 289.30 *m*/*z*, respectively; EGCG was [M − H]^−^ = 457.35 *m*/*z*; and ECG was [M − H]^−^ = 441.43 *m*/*z* (Figure 1B). Only caffeine was analyzed in the positive mass mode, which was [M + H]^+^ = 195.04 *m*/*z* (Figure 1C).

### 3.2. Effects of CE on Lung Morphology and Mucus Hypersecretion

**Figure 2:**
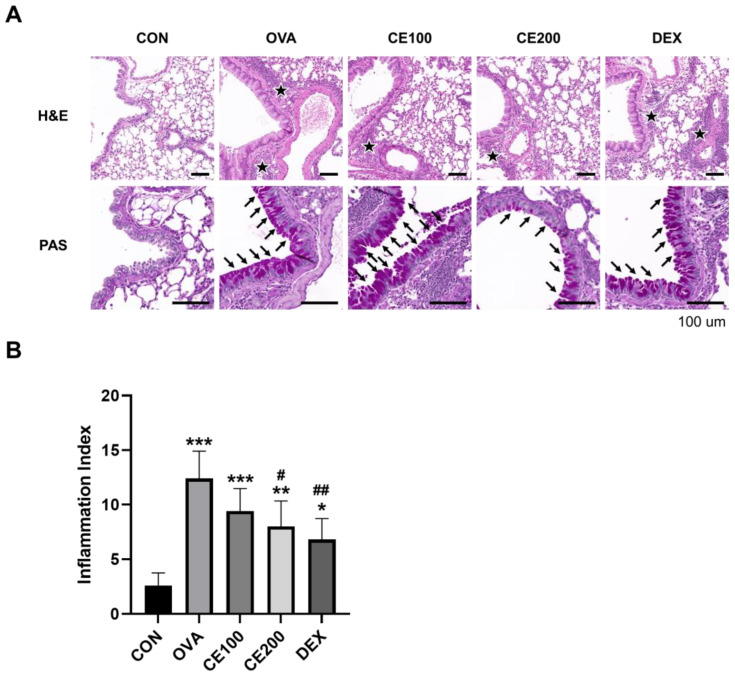
Effects of CE on lung morphology. (**A**) The lung tissue sections were stained with PAS and H&E. (**B**) Histopathological analysis on airway inflammation. CON: control group, OVA: OVA-induced asthmatic group, CE100: asthmatic + 100 mg/kg CE, CE200: asthmatic + 200 mg/kg CE, DEX: asthmatic + 5 mg/kg dexamethasone. Black stars indicate inflammatory cell infiltration. Black arrows indicate goblet cell proliferation and mucus hypersecretion. * *p* < 0.05, ** *p* < 0.01, and *** *p* < 0.001 compared to the CON group; # *p* < 0.05 and ## *p* < 0.01 compared to the OVA group. PAS: periodic acid–Schiff, H&E: hematoxylin and eosin, CE: *Camellia sinensis* (L.) Kuntze extract.

To examine the effects of OVA exposure and CE treatment on the morphology of lung tissue, H&E and PAS staining were performed, and inflammation-related alteration was measured (Figure 2). The results demonstrated increased immune cell accumulation in the OVA-induced asthmatic group compared to the control group tissues. However, decreased numbers of immune cells were detected in the DEX group, while the CE-treated group demonstrated a dose-dependent decrease in immune cells compared to the OVA-induced asthmatic group.

Mucus hypersecretion was measured using PAS staining. The results showed that the OVA-induced asthmatic group produced significantly more mucus than the control group, and the DEX- and CE-treated groups produced significantly less mucus than the OVA-induced asthmatic group. The CE-treated group exhibited a dose-dependent effect on the suppression of mucus production.

### 3.3. Effects of CE on Inflammatory Cell Numbers in the BALF of OVA-Induced Asthmatic Model Mice

**Figure 3:**
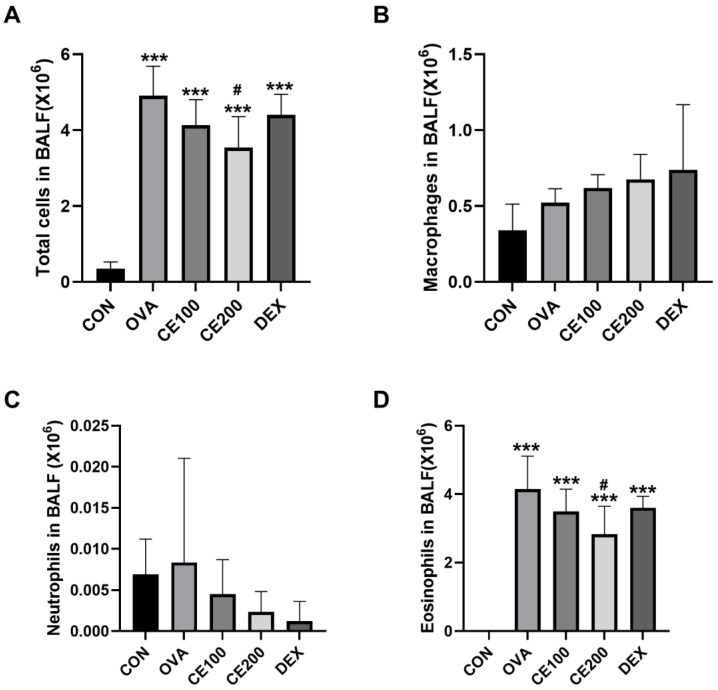
Inflammatory cell numbers in BALF in response to CE. Total cells (**A**), macrophages (**B**), neutrophils (**C**), and eosinophils (**D**) are shown. CON: control group, OVA: OVA-induced asthmatic group, CE100: asthmatic + 100 mg/kg CE, CE200: asthmatic + 200 mg/kg CE, DEX: asthmatic + 5 mg/kg dexamethasone. *** *p* < 0.001 compared to the CON group and # *p* < 0.05 compared to the OVA group. BALF: bronchoalveolar lavage fluid, CE: *Camellia sinensis* (L.) Kuntze extract.

Next, to confirm the effect of CE on inflammatory cell infiltration into lung tissue following the activation of hypersensitive immunological responses in OVA-induced asthma mice, the proportion and quantity of inflammatory cells in BALF were calculated using leukocyte differentiation. When compared to the control group, the OVA-induced asthmatic group showed a significant increase in eosinophils and total cells (*p* < 0.05) (Figure 3). Moreover, compared to the control group, the proportions of eosinophils and neutrophils were reduced following DEX treatment, although not significantly. The dose-dependent effects of CE treatment were analyzed for all cell numbers, especially comparing the CE200-treated group to the OVA-induced asthma model, where the numbers of total cells and eosinophils were significantly lower (Figure 3A,D).

### 3.4. Effects of CE on IL-4 and IgE Levels in the BALF of Asthmatic Mice

**Figure 4:**
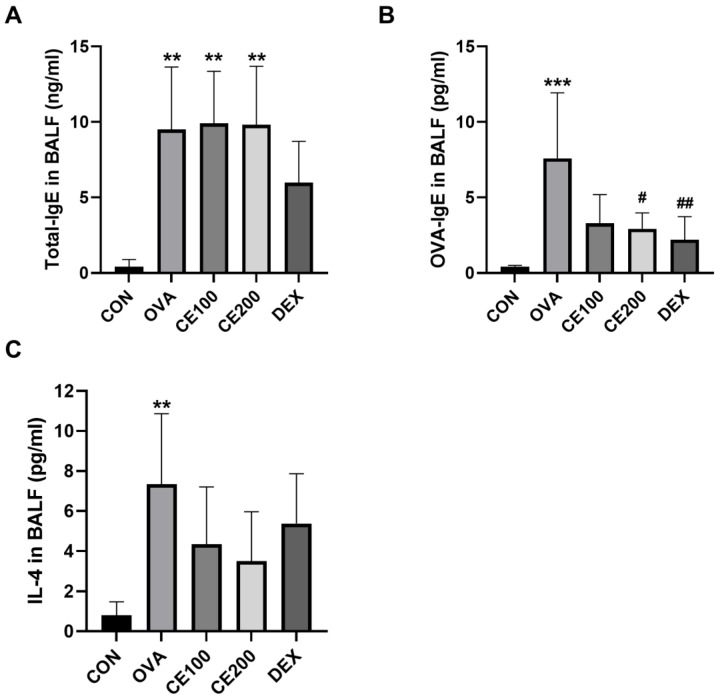
IgE expression and IL-4 production in bronchoalveolar lavage fluid (BALF) following treatment with CE. The concentrations of (**A**) total IgE, (**B**) OVA-specific IgE, and (**C**) IL-4 were assessed using an ELISA. CON: control group, OVA: OVA-induced asthmatic group, CE100: asthmatic + 100 mg/kg CE, CE200: asthmatic + 200 mg/kg CE, DEX: asthmatic + 5 mg/kg dexamethasone. ** *p* < 0.05 and *** *p* < 0.01 compared to the CON group; # *p* < 0.05 and ## *p* < 0.01 compared to the OVA group. BALF: bronchoalveolar lavage fluid, ELISA: enzyme-linked immunosorbent assay, CE: *Camellia sinensis* (L.) Kuntze extract.

BALF was subjected to ELISA to determine whether exposure to OVA increased the concentrations of IgE and IL-4 and whether CE had an inhibitory effect. A significant increase in IgE production level was detected in the OVA-induced asthmatic group compared to the control group (Figure 4). Moreover, the DEX group showed a lower level of total IgE and OVA-IgE levels compared to the OVA-induced asthmatic group, and a significant difference in OVA-IgE levels was observed. However, only the CE200-treated group demonstrated an effect of CE treatment on the levels of OVA-IgE, with a significant reduction compared to the levels observed in the OVA-induced asthmatic group (Figure 4B). Given the IL-4 levels detected in the BALF, we confirmed that the OVA-induced asthmatic group had substantially higher levels than the control group. Moreover, the IL-4 levels in the BALF were also lower in the CE-treated and DEX-treated groups compared to those in the OVA-induced asthmatic group, although not significantly.

### 3.5. Effects of CE on Antioxidant and Anti-Inflammatory Activities

**Figure 5:**
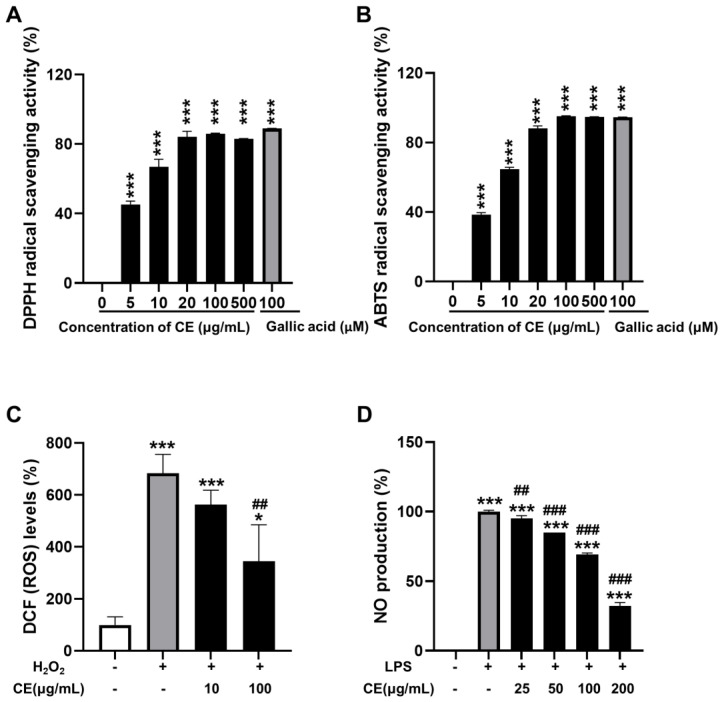
Effects of CE on anti-inflammatory and antioxidant activities. Anti-inflammatory activity detection data are shown for 2,2-diphenyl-1-picryl-hydrazyl-hydrate (DPPH) (**A**) and 2,2′-azino-bis-3-ethylbenzthiazoline-6-sulphonic acid (ABTS) (**B**), 2,7-dichlorodihydrofluorescein diacetate (DCFDA) (**C**), and scavenging activities in antioxidant activity detection and nitric oxide (NO) assay (**D**). * *p* < 0.05 and *** *p* < 0.001 compared to the LPS-non-treated group; ## *p* < 0.01, and ### *p* < 0.001 compared to the LPS-treated group.

Using an assay for DPPH, ABTS, and DCFDA radical scavenging activity, the antioxidative potential of CE was proven. Here, the results of the DPPH and ABTS radical scavenging activity assay revealed the antioxidant effect of CE (Figure 5A,B). Intracellular ROS levels were measured to investigate whether the protective effects of CE were related to anti-oxidant activities in H_2_O_2_-treated OP9 cells. As shown in Figure 5C, H_2_O_2_ increased the intracellular ROS levels significantly. This was significantly attenuated by pretreatment with CE (100 μg/mL). The radical scavenging activities of CE demonstrated dose dependence, with comparable values to the positive control (gallic acid) at a dose of 100 μg/mL.

To elucidate the role of CE in the LPS-mediated inflammatory response, Raw264.7 cells were pretreated with CE (100 μg/mL), and then, exposed to the LPS. LPS treatment significantly increased the levels of NO secreted into the culture medium. Additionally, a dose-dependent anti-inflammatory effect was shown for CE in the NO assay, while all doses showed significantly decreased values compared to those of the LPS-treated group (Figure 5D).

## 4. Discussion

Asthma is a chronic inflammatory airway disease with a worldwide prevalence and is responsible for 1 in 250 fatalities [28]. Previous research has employed corticosteroids to alleviate the symptoms of asthma. In the current study, DEX was used as a control medication to alleviate asthma symptoms. However, more effective agents are required due to issues relating to the long-term efficacy and safety of the currently available medications [29].

Green tea contains a substantial amount of polyphenols, including catechin, EC, GC, EGC, ECG, and EGCG, along with high amounts of caffeine [30]. Polyphenols in green tea provide photoprotective [31] and antioxidant [32] effects, and have shown a positive impact on asthma [33]. Several previous studies have examined the anti-allergic effects of polyphenols derived from green tea. In an experimental mouse model of OVA-induced allergic asthma, catechin showed potent anti-allergic activity by inhibiting histamine synthesis via the histidine-inhibitory effects of catechin isolated from Acacia catechu on an ovalbumin-induced allergic asthma model of decarboxylase inhibition [34]. In an experimental mouse model of OVA-induced food allergy, EC showed an anti-allergic effect via direct signaling to host receptors, leading to a local effect at the site of inflammation or a systemic effect mediated by EC-derived metabolites [35]. In a murine model of asthma in which airway inflammation was induced by toluene diisocyanate, EGCG exhibited anti-allergic properties by inhibiting the production of reactive oxygen species and matrix metalloproteinase-9 [36]. In an asthmatic mouse model, EGCG was demonstrated to reduce IL-10 levels while increasing IL-17A, BALF, and splenocyte levels, resulting in reductions in airway inflammation and hyper-responsiveness [37]. Therefore, in the current study, we investigated the effects of CE on an OVA-induced asthmatic disease model. Furthermore, several lines of evidence have indicated that the binding affinity of EGCG to specific proteins may explain its mechanism of action. In 2004, Tachibana et al. discovered that the cell surface 67-kDa laminin receptor (67LR) is the receptor of EGCG in an SPR study [38]. The binding of EGCG to the protein occurs at physiologically available concentrations and has been shown to mediate many of EGCG’s beneficial activities, including anti-allergic and anti-inflammatory activities [39].

Asthma is characterized as chronic airway inflammation, which includes mucus overproduction and inflammatory cell infiltration into the lungs [40]. Our results demonstrate that OVA-induced asthmatic disease causes the accumulation of immune cells and the production of mucus in the lungs, both of which were reduced by DEX treatment, with similar results observed in the CE-treated group, which demonstrated a dose-dependent trend. These results indicate that CE treatment can alleviate inflammation and mucus accumulation in asthmatic lung tissues.

Eosinophils play a role in the development of asthmatic inflammation, and an increase in eosinophils has been linked to asthmatic symptoms [41]. Furthermore, eosinophil infiltration is an indispensable indicator of airway inflammation and hyper-responsiveness, which can occur through an increase in eosinophil cationic proteins [42]. According to our findings, the number of total cells and eosinophils in BALF was enhanced by OVA treatment and alleviated by both DEX and CE treatments. These findings are supported by the anti-asthmatic effect of CE in relation to eosinophil release in BALF, with previous studies showing that increased numbers of eosinophils in the BALF are indicative of asthmatic disease [43].

Asthma is a type of allergic disease, and allergies occur through the interaction of antigens and antigen-specific IgE [44]. IL-4 is an important cytokine in Th2 inflammatory responses because it can induce the maturation of B cells and induce IgE conversion from IgG [45]. In our study, the amounts of IgE and IL-4 were significantly increased in the OVA-induced asthma model, while both DEX and CE treatments could effectively decrease the levels of IgE and IL-4. IL-4 has important pro-inflammatory functions in asthma including the induction of IgE, the expression of vascular cell adhesion molecule-1, the increase in eosinophil transmigration across the endothelium, mucus induction, and the differentiation of T helper type 2 lymphocytes, leading to cytokine release. Asthma is a complex genetic disorder that has been linked to polymorphisms in the IL-4 gene promoter and proteins involved in IL-4 signaling. The soluble recombinant IL-4 receptor lacks transmembrane and cytoplasmic activating domains and can therefore sequester IL-4 without mediating cellular activation [46]. Previous studies have shown that IL-4 is associated with mucus secretion, IgE production, and eosinophil activation [47]. Thus, considering these results and those of the current study, we hypothesized that IL-4 regulation is related to the effect of CE on asthmatic symptoms.

Green tea plants have antioxidant properties and pharmacological effects related to their ability to scavenge free radicals [18,48,49]. The crude pigment extract of *C. sinensis* (L.) Kuntze can scavenge DPPH radicals [49], while CEs can scavenge free radicals, thereby reducing oxidative damage to biomolecules [48]. The results of the current study demonstrate that CE effectively scavenged DPPH and ABTS radicals in vitro and prevented NO production, indicating that CE has lung-protective properties against hazardous substances.

Green tea does not cause severe side effects, even when consumed in excess, and several studies have demonstrated that it has beneficial effects on the health of various organs [50]. Green tea decreases oxidative stress [51] and relieves high-fat-diet-induced obesity and type 2 diabetes [52]. Moreover, in addition to preventing cardiovascular disease and cancer [53], green tea is also known to protect the nervous system [54]. Many studies have indicated that green tea is an asthma stimulant [55,56]. One of the primary components of green tea, EGCG, has been reported as an allergen in asthma cases [56]. Furthermore, in green tea-initiated asthma, EGCG boosts histamine release, exacerbating the symptoms [57]. Nonetheless, given its anti-allergic effects, green tea is commonly used in clinical practice for treating asthma [58,59]. Indeed, green tea has a positive protective effect against asthma and allergic rhinitis by preventing IgE expression in the cells of patients with asthma [58]. Theophylline, a licensed medicine made from green tea, is used for managing respiratory disorders such as asthma [60].

In conclusion, our research reveals that CE has a beneficial effect on asthma, which is related to the regulation of inflammatory cytokines. However, because this study only demonstrated the protective effect of CE on asthma, it is necessary to further examine the effect of EGCG on asthma. Additionally, green tea-based medications should be administered with caution in patients with asthma who react to EGCG as an allergen.

## Data Availability

The datasets generated during and/or analyzed during the current study are available upon request from the corresponding author.
